# Editorial: From sub-lexical to discourse-level effects in bi- and multilingual language processing

**DOI:** 10.3389/fpsyg.2025.1558057

**Published:** 2025-02-03

**Authors:** Katarzyna Jankowiak, Monika Połczyńska-Bletsos, Katarzyna Dziubalska-Kołaczyk

**Affiliations:** ^1^Faculty of English, Adam Mickiewicz University, Poznań, Poland; ^2^Department of Psychiatry and Biobehavioral Sciences, University of California, Los Angeles, Los Angeles, CA, United States

**Keywords:** bilingualism, multilingualism, language processing, cognition, cross-language interaction

This Research Topic delves into the dynamics of bi- and multilingual language processing, emphasizing the diverse influences of multilingualism across sub-lexical, lexico-semantic, and discourse levels. As linguistic diversity expands globally, the psycholinguistic effects of bilingualism and multilingualism continue to gather scholarly attention. The present contributions not only highlight the complexity of cross-linguistic interactions, but also stress the necessity of an interdisciplinary approach, bridging psychology, sociology, and linguistics. Each study advances our understanding of how multilingual individuals process language, adapt cognitively, and manage linguistic resources, ultimately enriching the field's broader perspective on the varied and evolving nature of multilingual cognition.

Geng et al. examined the processing of English-derived Japanese loanwords among Chinese learners of Japanese. Their study focused on factors such as familiarity, phonological similarity, context, and English proficiency. Familiarity was found to significantly reduce cognitive load, enhancing recognition, while the effect of phonological similarity diminished with higher Japanese proficiency. This finding suggests that advanced learners increasingly access Japanese meanings directly, bypassing reliance on English cues. The results underscore the importance of considering L1–L2 interactions when developing effective multilingual vocabulary resources.

Kędzierska et al. explored vowel perception in multilingual speakers of Polish, English, and Norwegian using event-related potentials. They examined how the mismatch negativity (MMN) response varies between a speaker's native language (L1, Polish) and non-native languages (L2 and L3/Ln). Results revealed that L1 elicited a stronger MMN response compared to L2 (English) and L3 (Norwegian), suggesting that language status modulates early auditory processing. This study enriches our knowledge of multilingual phonological perception and the roles of proficiency, dominance, and age of acquisition on phonemic discrimination.

Kim and Nam investigated the neural mechanisms underlying foveal word recognition through interhemispheric inhibition, using Korean visual stimuli. Their findings support the Split Fovea Theory, demonstrating that divided hemispheric processing reduces cognitive redundancy and enhances word recognition efficiency. While the study focused on monolingual word processing, its insights into hemispheric coordination and inhibitory control are relevant to bilingualism. These findings deepen our understanding of bilingual language control, cognitive resource management, and neural adaptability in multilingual individuals.

Laure and Armon-Lotem examined how Hebrew L2 bilinguals process templatic words, revealing that L1 mechanisms influence L2 word processing. Through a Hebrew rhyme judgment task, both Hebrew-native and Hebrew-L2 adults (both Semitic and non-Semitic L1s) were studied. Results indicated that Hebrew-L2 speakers utilize L1 patterns but show L2-specific adaptations, particularly in phonological or morphological awareness depending on their L1 background. Altogether, the findings highlight the influence of cross-linguistic transfer on L2 processing and provide insights into the role of non-linear morphology in bilingual language processing.

Wang et al. examined the effects of study-abroad experience (SAE) on Chinese (L1)–English (L2) interpreting students' translation skills. The study found that SAE participants translated more quickly but with more errors, indicating a speed-accuracy trade-off. Additionally, SAE participants demonstrated balanced bidirectional translation abilities, while non-SAE participants showed a preference for translating from L2 to L1. These findings suggest that SAE enhances cognitive flexibility and language-switching efficiency, pointing to the importance of immersive environments in interpreter training.

Baron et al. investigated grammatical gender processing in Spanish monolingual and Spanish–English bilingual children using eye-tracking. Testing children aged 5–10, they examined the use of gender cues in a visual world paradigm with grammatical and ungrammatical article-noun pairings. Results showed that bilinguals with greater English exposure were slower and less accurate in using gender cues than their monolingual peers. The findings highlight the impact of cumulative English exposure on grammatical gender processing and language control in bilingual children.

Fan and Wang investigated how L2 learners process formulaic sequences (FSs) during writing tasks with differing topic familiarity. The study distinguished internal FSs, which learners retrieve as whole units, from externally assembled FSs. The findings showed that high-proficiency learners more frequently retrieved and modified internal FSs, especially on familiar topics, indicating syntactic flexibility. In contrast, lower-proficiency learners assembled FSs word-by-word. These results suggest that L2 instruction should focus on internalizing FSs and promoting syntactic adaptability to enhance learners' writing fluency and accuracy, tailored to proficiency and topic familiarity.

Kul examined how Polish learners perceive reduced English forms, focusing on the effects of lexical context, phonetic reduction type, and musical background. The author found that lexical context and phonetic density significantly enhanced perception accuracy and speed, while musical training offered limited benefit, slightly improving reaction times but not accuracy. These findings suggest that language instruction should emphasize listening exercises featuring context-rich, naturally reduced speech patterns rather than idealized textbook clarity, helping learners navigate and understand authentic, connected spoken language more effectively.

Finally, Malarski et al. explored dialect use and style-shifting in the speech of Polish migrants in Norway, focusing on the acquisition of Norwegian (L3). Through sociolinguistic interviews in Oslo and Tromsø, the authors examined how first-generation migrants develop sensitivity to local dialects. Findings revealed that speakers vary in their use of regional features, with some acquiring dialectal forms similar to native speakers while others display less dialect use. The study offers valuable insights into multilingual dialect acquisition and sociolinguistic variation.

The articles in this Research Topic highlight the rich and varied landscape of multilingual language processing, spanning phonology, morphology, syntax, and discourse. Collectively, they underline the role of cognitive and linguistic factors, including proficiency, cross-linguistic influence, and immersion, in shaping language processing. Findings reveal how language status affects phonemic processing, how immersive experiences refine translation skills, and how bilingualism influences grammatical gender sensitivity and formulaic sequence usage. Ultimately, this body of work illustrates the adaptive and dynamic mechanisms of multilingual cognition, providing a nuanced understanding of how multilingual individuals navigate and manage complex linguistic resources across various contexts.

